# Revealing the Reactivity of Individual Chemical Entities in Complex Mixtures: the Chemistry Behind Bio-Oil Upgrading

**DOI:** 10.1021/acs.analchem.2c00261

**Published:** 2022-05-16

**Authors:** Diana Catalina Palacio Lozano, Hugh E. Jones, Remy Gavard, Mary J. Thomas, Claudia X. Ramírez, Christopher A. Wootton, José Aristóbulo Sarmiento Chaparro, Peter B. O’Connor, Simon E. F. Spencer, David Rossell, Enrique Mejia-Ospino, Matthias Witt, Mark P. Barrow

**Affiliations:** † Department of Chemistry, University of Warwick, Coventry CV4 7AL, U.K.; ‡ Molecular Analytical Science Centre of Doctoral Training, University of Warwick, Coventry CV4 7AL, U.K.; § Laboratorio de Espectroscopía Atómica y Molecular (LEAM), Universidad Industrial de Santander, Bucaramanga 678, Colombia; ∥ Centro de Materiales y Nanociencias (CMN), Universidad Industrial de Santander, Bucaramanga 678, Colombia; ⊥ Bruker Daltonics GmbH & Co. KG, Bremen 28359, Germany; # Instituto Colombiano del Petróleo (ICP-Ecopetrol), Piedecuesta 681019, Colombia; ¶ Department of Statistics, University of Warwick, Coventry CV4 7AL, U.K.; ∇ Department of Economics & Business, Universitat Pompeu Fabra, Barcelona 08005, Spain

## Abstract

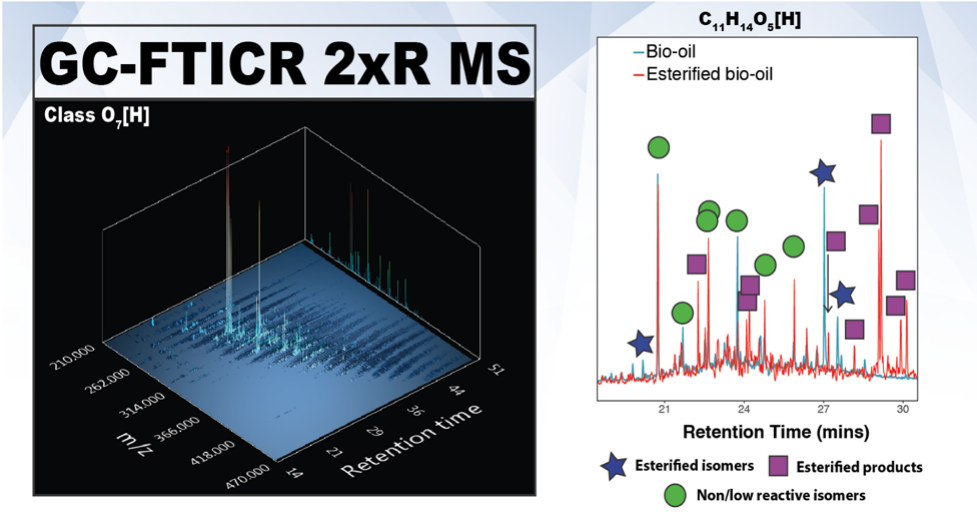

Bio-oils are precursors for biofuels but are highly corrosive necessitating further upgrading. Furthermore, bio-oil samples are highly complex and represent a broad range of chemistries. They are complex mixtures not simply because of the large number of poly-oxygenated compounds but because each composition can comprise many isomers with multiple functional groups. The use of hyphenated ultrahigh-resolution mass spectrometry affords the ability to separate isomeric species of complex mixtures. Here, we present for the first time, the use of this powerful analytical technique combined with chemical reactivity to gain greater insights into the reactivity of the individual isomeric species of bio-oils. A pyrolysis bio-oils and its esterified bio-oil were analyzed using gas chromatography coupled to Fourier transform ion cyclotron resonance mass spectrometry, and in-house software (KairosMS) was used for fast comparison of the hyphenated data sets. The data revealed a total of 10,368 isomers in the pyrolysis bio-oil and an increase to 18,827 isomers after esterification conditions. Furthermore, the comparison of the isomeric distribution before and after esterification provide new light on the reactivities within these complex mixtures; these reactivities would be expected to correspond with carboxylic acid, aldehyde, and ketone functional groups. Using this approach, it was possible to reveal the increased chemical complexity of bio-oils after upgrading and target detection of valuable compounds within the bio-oils. The combination of chemical reactions alongside with in-depth molecular characterization opens a new window for the understanding of the chemistry and reactivity of complex mixtures.

## 1 Introduction

Biomass is considered an alternative renewable resource for the production of biofuels and valuable chemicals.^1^ To produce biofuels from lignocellulosic materials, it is necessary to depolymerize the feedstock. A technology widely used is fast pyrolysis.^2^ The fast pyrolysis oil, often call bio-oil or tar, has undesirable characteristics such as high amount of reactive oxygenated compounds, high water content, low solubility in water, and immiscibility with petroleum-derived fuels;^3^ it is clear that further upgrading is needed to produce higher-value fuels or chemicals.^3^ Catalytic esterification is widely used for this purpose.^4^ In summary, esters are formed by the reaction of carboxylic acids with alcohols in the presence of a strong acid, effecting the elimination of a water molecule.^4^ The acetalization of aldehydes, ketones, and sugars has also been reported as a main reaction between pyrolyzed bio-oil and alcohols.^5^

Given the high complexity of bio-oils, chemical characterization is inherently challenging. Traditionally, gas chromatography mass spectrometry (GC–MS) has been used to perform qualitative and quantitative analyses of the low-molecular-weight composition of bio-oils.^6^ However, GC–MS does not allow detailed analysis of bio-oil compositions due to its limited peak capacity and selectivity compared to two-dimensional chromatography (GC × GC MS).^7^ Currently, studies using GC–MS have identified up to 166 volatile compositions.^8^ Other hyphenated techniques, such as GC × GC MS, provide increased chromatographic separation, allowing a larger number of components with similar co-elution times to be resolved.^8^

Complex mixtures such as petroleum-related compounds and bio-oils can also be analyzed by ultrahigh-resolution MS.^9−12^ The high mass accuracy and high resolving power achieved by Fourier transform ion cyclotron resonance mass spectrometry (FTICR MS) allows a unique molecular formula to be assigned to each detected composition of a complex sample without any prior fractionation.^9^ The sample will comprise thousands of elemental compositions (molecular formulae), which can be assigned as C_c_H_h_N_n_O_o_S_s_ where c, h, n, o, and s represent the number of carbon, hydrogen, nitrogen, oxygen, and sulfur atoms, respectively.^13^ The detailed elemental compositions are then categorized by heteroatomic class (e.g., the N_n_O_o_S_s_ content of the molecular formula), carbon number, and number of double bond equivalents (DBEs).

Despite the high performance of FTICR MS, isomers cannot be resolved on the basis of *m/z* alone. Thus, when direct infusion experiments are performed, multiple isomers of the same, unique elemental composition may be ionized and be observed at a single *m/z*. The isomers may have very different properties and reactivities, however, and it is important to gain greater insights into the composition of complex mixtures, including contributions from different functional groups. To separate isomeric species, mass spectrometric techniques are typically coupled with chromatographic systems such as ultra-high performance supercritical fluid chromatography (SFC),^14^ GC,^15−18^ ion mobility spectrometry,^19^ SFC,^20,21^ and liquid chromatography.^22^

In this study, a new analytical method is presented to gain information on the functional groups in complex mixtures and their role in reactivity under esterification conditions. In short, the isomeric information of a pyrolysis bio-oil and its esterified product was obtained experimentally by using a combination of GC, atmospheric pressure chemical ionization (APCI), and FTICR MS (i.e., GC–APCI–FTICR MS). Custom software incorporating a peak picking algorithm was then used to compare isomeric contribution before and after esterification conditions, which allowed us to identify the isomeric species that reacted during the upgrading process. By taking into account that esterification transforms carboxylic acids and carbonyl groups into esters, it was possible to classify isomers that might contain carboxylic groups and the compositions containing ester groups.

We believe that the method here proposed is more efficient and robust, as instead of assessing individual compounds, we classify simultaneously the diverse chemical entities based upon their reactivities. Using this new approach, it was possible to characterize the highly complex mixtures and determine the presence of reactive chemicals within the crude bio-oil.

## 2 Experimental Section

### 2.1 Sample Description

A raw bio-oil obtained from the pyrolysis of a mixture of softwood material and its esterified product were analyzed. A detailed information of the pyrolytic and esterification conditions can be found elsewhere.^23^ The crude bio-oil was subjected to esterification/acetalization with *n*-butanol in the presence of H_2_SO_4_/dehydration to produce an upgraded product. Hereafter referred to as the “esterified bio-oil,” this upgraded product results from the combined esterification of carboxylic acids to esters and acetalization of aldehydes and ketones into acetals. A reaction yield of 68% over the crude bio-oil was reported.^22^

### 2.2 GC–APCI–FTICR MS

The analysis of the samples was performed using GC–APCI–FTICR MS. Briefly, a GC 450 (Bruker Daltonik GmbH, Bremen, Germany) was coupled to a GC–APCI II ion source, and the ions were detected by a 7 T solariX 2xR FTICR mass spectrometer (Bruker Daltonik GmbH, Bremen, Germany). The samples were dissolved in acetone to a final concentration of 3 and 5 ppm (bio-oil and esterified bio-oil, respectively), and 1 μL was injected into a 30 m DB-5 column (0.25 mmID, 0.25 μm). Helium was used as the carrier gas, and the oven temperature was programmed from 60 to 300 °C at a heating rate of 6 °C min^–1^ and held at 300 °for 9 min. The mass spectra were acquired with a detection range of *m/z* 107–3000 and a data set size of 2 MW, resulting in a detection time of 0.52 s. Quadrupolar phase detection was employed by a solariX 2xR instrument. This technique affords the rapid scan rate required for hyphenated data sets. The data sets, acquired in the magnitude mode, exhibited a resolving power of 302,000 at *m/z* 200. A single mass spectrum was acquired every 0.9 s, and therefore up to 3100 mass spectra comprise the total ion chromatogram (TIC) of each sample. The TICs can be found in Supporting Information (Figure S1).

### 2.3 Data Processing

The elemental composition corresponding to the protonated species, [M + H]^+^, of hexamethylcyclotrisiloxane (D3), with an *m/z* of 223.06345,^24^ was used for internal single-point calibration of the data sets using DataAnalysis. The data sets were further processed and analyzed using in-house software named KairosMS.^25^ Briefly, a peak list was generated for each data set, containing the absolute intensity and retention time of each ion, using DataAnalysis 4.2 (Bruker Daltonik GmbH, Bremen Germany). The mass lists were individually opened in KairosMS, where a recalibration and intensity filter are applied, and a final unique mass list per sample are exported. The unique mass lists were assigned using Composer 1.5.6 (Sierra Analytics Inc., Modesto, CA, USA). An internal walking recalibration with abundant homologous alkylated compounds corresponding to O_2_[H] were applied in Composer prior to assignment of molecular formulae. Each elemental composition was assigned with a maximum formula of C_200_, H_1000_, O_20_, N_3_, S_3_ and maximum DBE of 40 with up to 1.5 ppm error. Molecular compositions detected in the bio-oil and the esterified bio-oil were assigned with a final root-mean-square error of 0.103 and 0.168 ppm, respectively. The peak assignments were exported from Composer and imported back into KairosMS to merge the processed data and assignments. The information of the individual assignments and their retention time can then be visualized in KairosMS by a variety of plots commonly used in petroleomics.^26^ Finally, the correlation of each elemental contribution with absolute intensity and retention time was analyzed using an algorithm included in KairosMS, providing peak detection within each extracted ion chromatogram (EIC). The peak picking criteria are described in Section 2 in Supporting Information (Figures S2 and S3).

## 3 Results and Discussion

### 3.1 Sensitivity and Resolving Power of GC–FTICR MS

In this work, up to 3100 mass spectra comprise each TIC. An animation of the evolution of the mass spectra with the retention time can be found in Movie S1 in Supporting Information. A shift toward higher *m/z* was observed as the retention time increased. As expected, compositions with higher *m/z* values showed increased boiling points and were detected at longer retention times.

**Figure 1:**
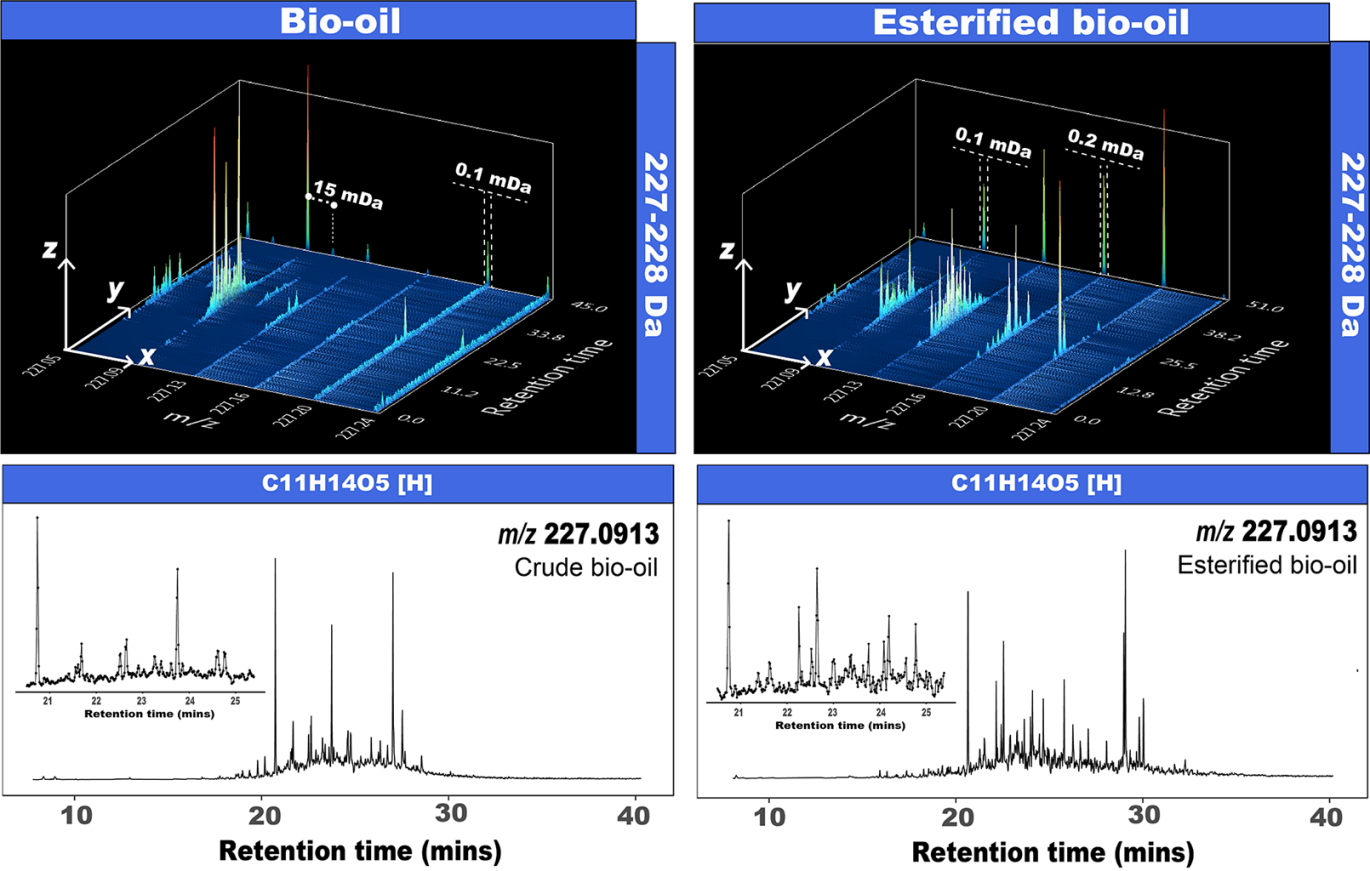
Above: three-dimensional plots illustrating the assigned EICs within a 1 Da window centered at *m*/*z* 227. Below: EICs for the composition C_11_H_14_O_5_[H] of the crude bio-oil (left) and esterified bio-oil (right). Each data point of the EIC was obtained with a time separation of 0.9 s, as illustrated by the distributions shown in the insets.

To illustrate the high complexity and capabilities of ultrahigh-resolution MS hyphenated with GC, a detailed analysis of the assigned EICs spanning a window, with an *m/z* width of 1 and centered at *m/z* 227, is shown in Figure 1. Details of the assignments made can be found in Table S1. In Figure 1, the *xy* and *zy* planes show the evolution of the individual compositions with the retention time, and the projections in the *xz* plane represent the molecular assignments without chromatographic separation (similar to what would be observed by direct infusion). The ultrahigh performance achieved by FTICR MS, coupled to the ability of the structural isomer separation by GC, allows the monitoring of the elution of species corresponding to the eight elemental compositions within the mass range, as shown in Figure 1. As shown in this figure, the EICs of species separated by only 15 mDa, corresponding to C_15_H_14_O_2_[H] and C_11_H_14_O_5_[H], were baseline resolved. The data points that defined the EICs are expanded in 0.1 and 0.2 mDa with a maximum standard deviation of 2.4 × 10^–5^, demonstrating that species with a mass difference as low as 0.2 mDa can be resolved at *m/z* 227. The ultrahigh resolution achieved by a solariX 2xR instrument (see the Experimental Section) then allows the resolution of very narrow mass differences. Examples include CH_4_ versus O (36.4 mDa), CH_2_ versus N (12.6 mDa), C_3_ versus SH_4_ (3.4 mDa), and ^13^C versus CH (4.5 mDa). Compositions such as C_10_H_12_O_3_ versus C_9_H_10_O_3_^13^C_1_[H], with a mass split of 4.47 mDa, were commonly found within the bio-oil and its esterified product. A mass resolving power of >145,000 is needed to resolve this mass split at *m/z* 650.

The scan rate facilitated using the mass spectrometer operating with 2ω detection permits a duty cycle of about 1 Hz. Thus, a mass spectrum is acquired every 0.015 min, and multiple data points corresponding to each individual composition can be collected during the GC–FTICR experiments. For instance, 1711 and 1522 data points were detected for the EICs for the molecular formula C_11_H_14_O_5_[H] in the esterified and crude bio-oil, respectively. According to the EICs displayed in Figure 1, up to 31 and 57 isomers of the composition C_11_H_14_O_5_[H] were detected in the bio-oil and esterified bio-oil samples, respectively, and structural isomers eluting from the column with a retention time difference of 0.098 min can be baseline resolved (see related examples in Figure S4).

### 3.2 Compositional Analysis

**Figure 2:**
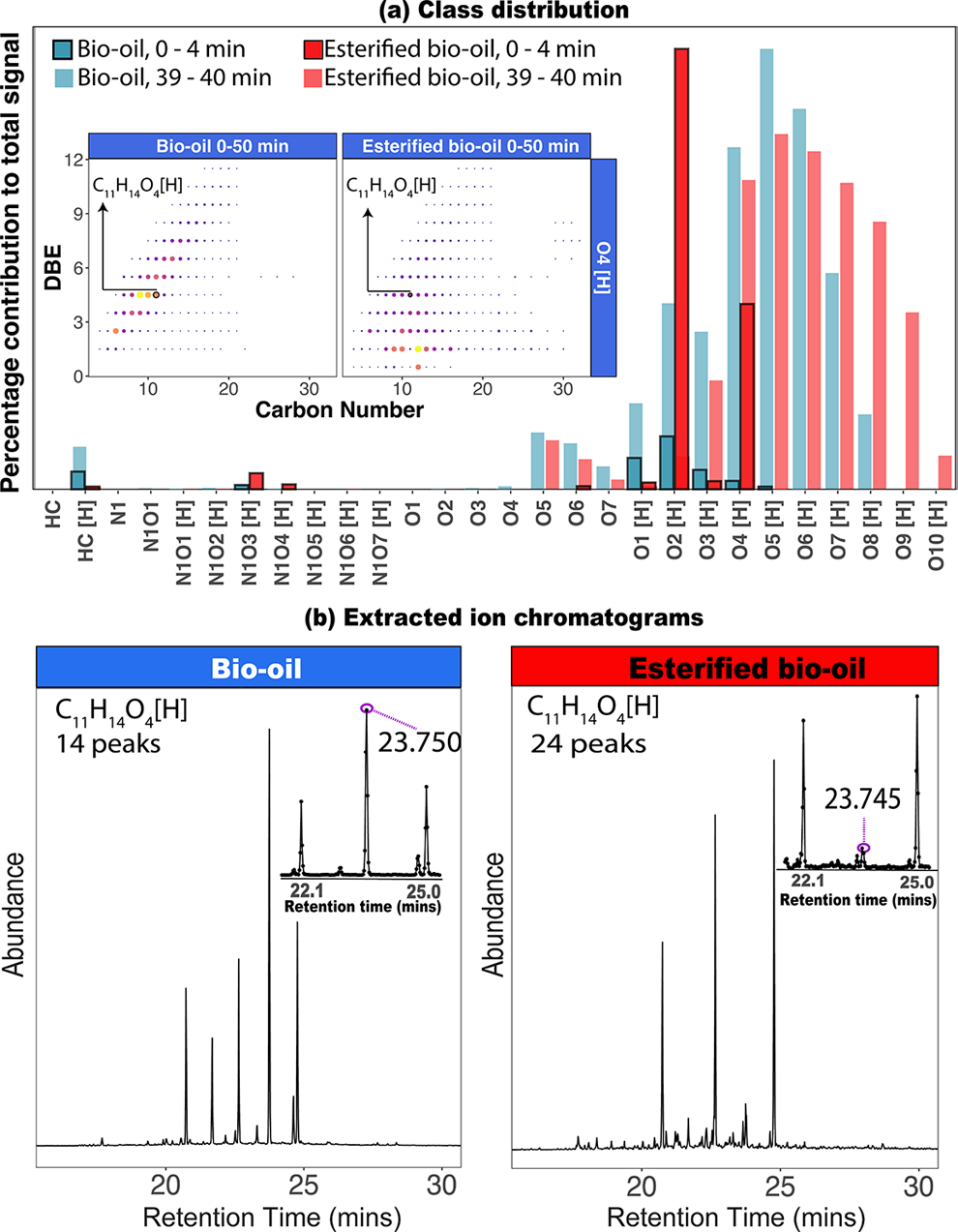
(a) Class distribution and DBE (inserts) plots of the bio-oil and the esterified bio-oil at selected retention times. (b) EICs of the composition C_11_H_14_O_4_.

The mass spectra of the volatile chemicals in the crude bio-oil and its esterified product comprise 1743 and 2531 assignable EICs (i.e., therefore the number of elemental compositions), respectively, each with assigned molecular formulae detected in the mass range between *m/z* 107–800. As shown in Figure 2, the heteroatomic class distributions change as a function of the retention time. For instance, species with low oxygen-content, low DBE, and low carbon number elute from the column at shorter retention times, while highly oxygenated species elute at later retention times. The evolution of the class distribution with the retention time can be found in Movie S1.

The class distribution in Figure 2 shows a higher relative abundance of the protonated species [M + H]^+^ upon ionization by positive-mode APCI; protonated species are denoted by the inclusion of “[H]” in the heteroatom class, while radical ion species do not include this tag. A total of 456 and 502 EICs were observed as both protonated and radical ion forms, for the bio-oil and the esterified bio-oil respectively, representing about 14% of the assigned molecular compositions. A comparison of the EICs of the protonated and radical ions (see related examples Figures S5 and S6) shows that most of the isomers preferentially form abundant protonated species. It is important to consider that unlike electrospray ionization, APCI usually produces some degree of fragmentation, which can limit detection of some more labile radical ion species.

As can be seen in Figure 2, a shift toward higher oxygen content was observed after the reaction. In general, the boiling points of esters are lower than the corresponding acid. For example, ethyl formate has a boiling point of 54.6 °C while that of formic acid is 100.5 °C.^27^ The increased volatility of the medium and heavy compositions of bio-oil after esterification has been reported.^28^

As can be seen in Figure 2, 14 and 24 peaks were detected within the compositions corresponding to C_11_H_14_O_4_[H] before and after the reactions, respectively. Thus, many different structural arrangements (isomers) were eluted at different retention times. The data presented here shows that bio-oils are complex not simply because of the large number of poly-oxygenated compositions (i.e., a large number of EICs) but because of the large number of isomeric contributions found within the samples too. Oxygen-containing functional groups such as organic acids can be converted into more stable esters after esterification. Simultaneously, it is possible to convert reactive aldehydes into more stable acetals by acetalization of aldehydes with alcohol.^28^ Thus, the formation of esters and acetals after the reaction increases the isomeric complexity while simultaneously introducing many lower boiling point oxygen-containing species. As a result, the total relative abundance of the detected oxygen-containing species increases. This explains the increased number of isomers detected in each EIC in Figure 2, and the shift toward higher oxygenated elemental molecular compositions after esterification.

When considering the total count of EICs detected, a predominance of even carbon numbers compared to odd carbon numbers was observed among the esterified bio-oil components at higher carbon numbers (see Figure S7). This is in agreement with the previous literature, where the distribution of esterified long-chain *n*-fatty acids and higher-molecular-weight diacids have been characterized by an strong even/odd predominance.^29−31^ The even to odd predominance of fatty acids in the range from C_12_ to C_32_ is typical for terrigenous higher land plants.^32,33^

It is interesting to note that some peaks (see Figure 2) such as at 13.047 min and 23.750 min for C_6_H_8_O_4_[H] and C_11_H_14_O_4_[H], respectively, were not detected after esterification (also see Figures S8–S10). The absence of the signal at those particular retention times indicates the apparent complete reaction.

### 3.3 Potential of Targeted Analysis of Chemicals in Bio-Oils

As shown in the following sections, the comparison of elution times and relative abundances of each isomeric structure detected in each sample is fundamental for targeted analysis and future quantification.

**Figure 3:**
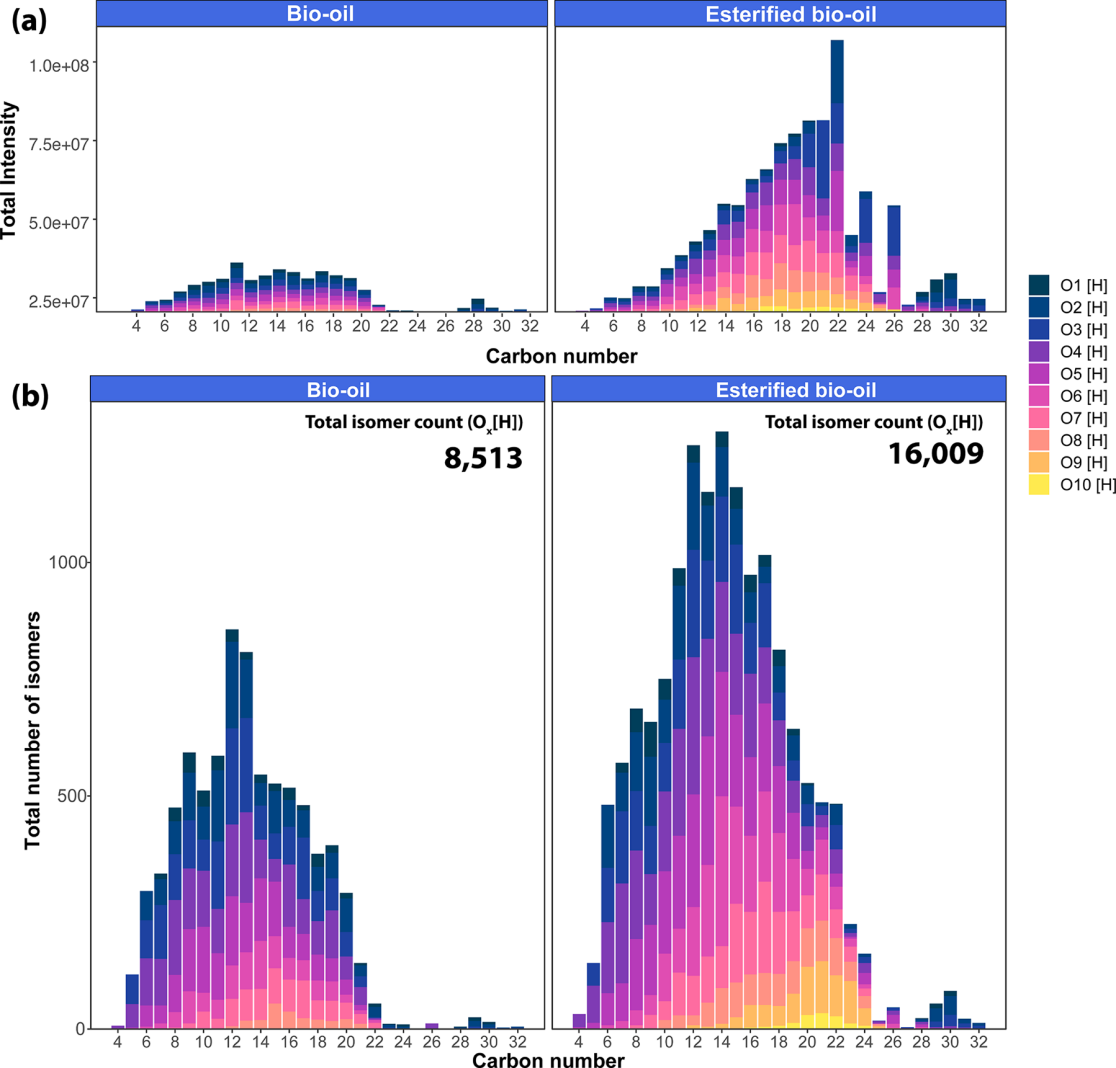
Distribution of the total number of isomers for the bio-oil sample and its esterification product (esterified bio-oil), counted by carbon number and DBE for the protonated oxygenated species.

The peak detection criteria allowed the determination of 10,368 (8513 protonated) and 18,827 (16,009 protonated) structural isomers in the bio-oil and the esterified bio-oil, respectively, including isomers detected in EICs corresponding to isotopologues. As shown in Figure 3, the distribution of the esterified isomers was shifted toward higher carbon number as a consequence of the catalyzed esterification of carboxylic acids, ketones, and aldehydes. Additionally, the total isomer count sorted by DBE indicates a shift toward a detection of isomers with lower DBE after esterification. This indicates the formation of acetals from aldehyde or ketone functional groups (see Figure S11), where a double bond between carbon and oxygen in a carbonyl group is converted to a single bond between the two.

One of the main limitations in targeted and quantitative analysis remains in the availability of MS spectra of bio-oil compositions in databases.^34^ The list with perhaps the largest number of identified compositions in bio-oils was provided by Staš et al. in 2014.^35^ The database comprises 370 identified chemicals by GC–MS and GC × GC methods. By comparing the lists of chemicals provided by Staš et al. and Branca et al.^36^ with our GC FT–ICR MS data, it was possible to determine 84 common elemental compositions (see xlsx spreadsheet in Supporting Information). Greater numbers of isomers were detected within the EICs corresponding to compositions such as C_6_H_10_O_5_ (which may represent levoglucosan), C_8_H_8_O_3_ (which may represent vanillin), and C_8_H_10_O_1_ (which may represent 2–3 ethylphenol), showing the high-performance of GC–APCI–FTICR MS.

There is a noticeable difference among the mass range of the compositions reported by GC–MS and GC × GC methods in comparison with GC–FTICR MS data obtained in this work. About 65% of the compositions summarized by Staš are in a range of *m/z* 90 to 130. In contrast, only 3 and 2.4% of the molecular compositions detected in the bio-oil and its esterified sample, respectively, were detected in this mass range. This indicates the increased sensitivity of detection achieved by solariX 2xR FTICR MS, leading to the observation of chemicals of higher mass than cited in the previous literature. It is also clear that in comparison with GC–FTICR MS (here, tuned for *m/z* 107 and higher), GC–MS and GC × GC can be more amenable to the detection of lower masses with higher sensitivity. Therefore, the methods can be valuable complementary analytical techniques for a comprehensive analysis of bio-oils.

As shown in Figure 3, isomeric species with carbon number up to 32 were detected within the bio-oil compositions. The total number of isomers in the samples increased with carbon number (*C*) for species with *C* ≤ 13 and decreased at higher carbon content. It is well known that the number of isomers increases rapidly with carbon number as a consequence of the high number of possible structural arrangements.^37^ The boiling point of a predominantly hydrocarbon molecule generally increases as the number of atoms increases. Therefore, due to their low volatility, high carbon number species are not well separated by retention time (see related example in Figure S12) and may not be amenable to GC-based separation approaches. An alternative separation method to GC to study high-molecular-weight components in bio-oils may be SFC.^14,38^

A second high-carbon number distribution (C_26_–C_32_) was found for the bio-oil and esterified bio-oil samples (see Figure 3). These compositions can be attributed to steroids, previously identified by Pakdel and Roy in 1996,^39^ in various biomass-derived vacuum pyrolysis oils. Steroids are not only valuable chemicals used in pharmaceutical applications but can also be biomarkers or contaminants. The possible presence of stigmasterol (C_29_H_48_O_1_), (−)-cholesterol acetate (C_29_H_48_O_2_), cholesta-3,5-dien-3-yl acetate (C_26_H_49_O_2_), and camellenodiol (C_29_H_46_O_3_) can be explored in future targeted analyses (see Figure S13).

Potentially valuable chemicals in bio-oils can be explored by a targeted approach using GC–FTICR MS. It is important to consider that the positive identification of the individual isomers is laborious due to the large number of analytes and the lack of authentic standards with known response factors for identification and quantification. The exact mass data provided by GC–FTICR MS can be used in future studies as an additional scoring factor for chemical identification. Such analysis, however, lies outside of the scope of this paper.

### 3.4 Detailed Assessment of Compositional Changes during Esterification

The difficulty in understanding how the esterification affects the composition of the esterified bio-oil arises from the many possible functionalities that may be present in the arrangement of thousands of compositions, combined with poorly understood reaction routes within complex mixtures, such as bio-oils, under the esterification conditions.^4^

Chemical reactions such as esterification can produce multiple organic acid esters because of the reaction of one or multiple acid groups within a molecule. For instance, the esterification reaction of succinic acid with ethyl alcohol can produce mono-ethyl succinate and di-ethyl succinate with yields that vary depending on the reaction system conditions.^40^ Independently of the esterification yield, each product is likely to present a distinctive boiling point, which differs from the boiling point of the reactive organic acid. A direct comparison of the retention times of individual molecular isomers across each EIC can therefore help understand the reaction behavior of the isomers during the esterification of the bio-oil.

To perform this analysis the following assumptions and considerations were made: (a) the main reported reactions that can take place under esterification conditions are the formation of molecules containing ester and acetal functional groups from carboxylic acids and aldehydes or ketones, respectively (thus, reactions such as transesterification, etherification, acetalization, dehydration of sugars, degradation of furans, and conversion of phenolics are not considered),^4^ (b) due to the different sample concentrations, a quantitation of the partial esterification is not performed in this work, (c) the chromatographic separation reduces the suppression effects (compared to direct infusion) during the ionization of the analytes,^41^ and therefore, similar ionization efficiencies for the same analyte in both samples are expected, (d) the comparison of the isomeric composition was performed between the individual EICs of protonated compositions that are common in both samples, (e) a retention time difference of up to 3 s is permitted in the peak matching when comparing isomeric distributions between the bio-oil and esterified bio-oil samples (see related examples in Figure S14), and (f) the reactivity criteria will be defined as follows:

- Highly reactive isomers: individual isomeric species detected only in the raw bio-oil.
- New isomeric products: isomers detected only after reaction.
- Low/non-reactive isomers: isomeric species detected in both samples. That is, moieties observed at the same retention times and within the same EICs for the bio-oil and esterified bio-oil samples.

**Figure 4:**
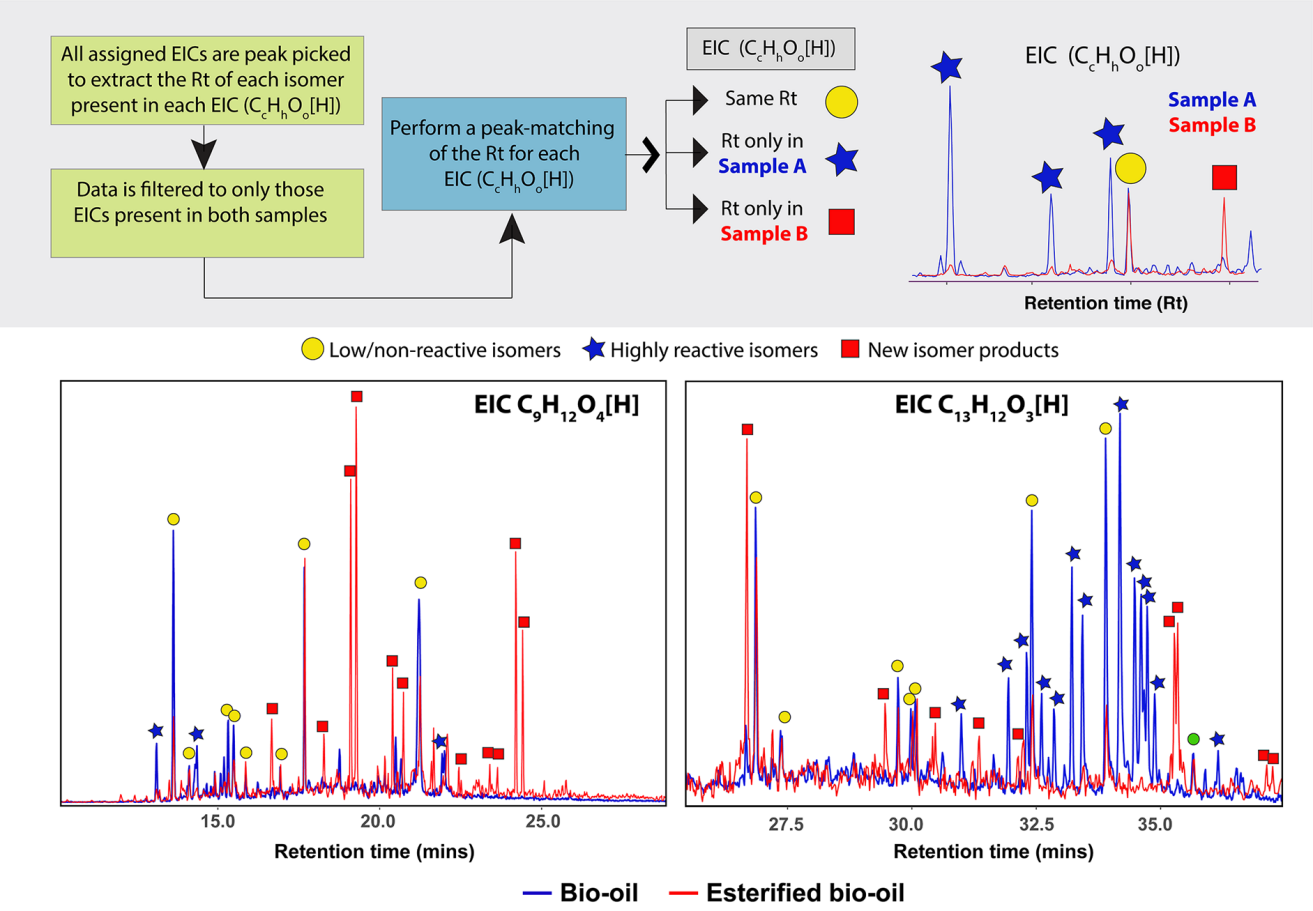
Top; a schematic representation of the peak matching of the retention time (Rt) performed within each EIC of a sample A and B. In this work, the reactivity was defined according to the matching between the Rt of the isomers in the bio-oil and its esterified product as follows: highly reactive isomers only detected in the bio-oil, new isomeric products if detected only after the reaction, and low/non-reactive species if the peak is detected in both samples. Bottom: examples of the peak matching of the EICs for C_9_H_12_O_4_[H] and C_13_H_12_O_3_[H].

A schematic of the approach can be seen in Figure 4.

As a consequence of the high complexity of the esterification and the large number of molecular compositions involved in the reaction, it is not possible to identify the products from each reactant. Additionally, the total number of reactive carbonyl/carboxyl functional groups of each isomer cannot be determined. Isomers classified as highly reactive contain at least one reactive carboxyl or carbonyl group. Similarly, the new isomeric products can correspond indistinctively to mono- or poly-esterification of an unknown reactant. Peaks defined as low/non-reactive isomers (isomers detected in both samples) may correspond to three distinctive reactions: partial esterification if the relative intensity is reduced after esterification, isomeric products of the reaction that are already present in the bio-oil (increased relative abundance after esterification), or non-reactive isomers if the relative intensity is similar in both samples. Some related examples are shown in Figure S15. Due to the complexity of the evaluation of the relative abundance of the many common isomers, our current method does not include the distinction of those reactions. Thus, those reactions are defined here just as low/non-reactive isomers.

Bearing in mind these assumptions, it is possible to estimate the total number of isomeric structures representing high reactivity under the esterification conditions. Consider, for example, the EICs of C_9_H_12_O_4_[H] and C_13_H_12_O_3_[H], as shown in Figure 4. The isomers denoted with a blue star were not detected in the esterified sample. Therefore, these highly reactive species may contain carboxylic acid, ketone, or aldehyde functional groups that were esterified. Additionally, the isomers denoted with a red square were only detected after reaction, which indicates new isomers produced after upgrading (reaction products). Finally, the peaks detected in both samples (yellow circle) correspond to low- or non-reactive isomers.

Isomeric comparisons were performed for compositions found within both the bio-oil and the esterified bio-oil, comparing the relevant pairs of EICs. The results are summarized by heteroatom class in Table 1. The high variety of products in all heteroatomic classes can be explained by many possible combinations of oxygen-containing functionalities that can result following the upgrading reaction. Thus, when an individual composition represents multiple functionalities such as amino, carboxyl, carbonyl, and aldehyde groups, the esterification tends to produce mixtures of isomeric structures.^42^ Among 13,790 total isomers that can be compared within the samples, 30.4% correspond to highly reactive species that may contain at least one carboxylic acid, aldehyde, or ketone functional group that was effectively transformed under esterification conditions and 26.3% remain after the reaction due to the low- or non-reactivity of these chemicals under these conditions.

**Table 1:** Total Number of Isomers and the Percentage (%) Observed Per Class^a^

class	highly reactive isomers	low/non-reactive isomers	new isomeric products
O_1_[H]	121 (20.3)	175 (29.4)	300 (50.3)
O_2_[H]	595 (30.6)	516 (26.6)	831 (42.8)
O_3_[H]	863 (34.5)	532 (21.2)	1110 (44.3)
O_4_[H]	762 (27.9)	654 (23.9)	1317 (48.2)
O_5_[H]	476 (21.6)	576 (26.1)	1153 (52.3)
O_6_[H]	281 (18.8)	304 (20.4)	906 (60.8)
O_7_[H]	331 (31.2)	243 (22.9)	488 (46.0)
O_8_[H]	151 (34.1)	69 (15.6)	223 (50.3)
total	3580	3069	6328

a Highly reactive isomers are detected only in the bio-oil sample. Low/non-reactive are isomers detected in the bio-oil, and the esterified bio-oil, and new isomeric products are isomers detected only in the esterified bio-oil.

It is important to note that the data presented in Table 1 are based upon a database generated from the GC–FTICR MS data and represent a simpler way to summarize the results. The database contains fuller information, such as the molecular formula, the retention times, and the relative abundance of each isomer detected, along with the commonality between the bio-oil and its esterified isomers. This database can be used in future studies for data modeling that inform bio-oil production and upgrading, with a view to further advances in biofuel development.

Thus, although targeted analysis can help identify individual valuable chemical species within bio-oils, a direct comparison of the isomeric distribution, as proposed here, of bio-oils under different upgrading conditions can reveal remarkably detailed information of the reactivity of multiple chemicals that comprise the bio-oil. For instance, the effectiveness of different pyrolysis conditions, chemo-selectivity of different reaction methodologies, and multistep synthetic strategies can all be better understood.

## 4 Conclusions

A pyrolyzed softwood bio-oil and its upgraded (esterified) product have both been characterized using a novel combination of experimental and data processing approaches. Through the usage of GC–APCI FTICR MS and a new method for the data analysis, detailed assessment of the isomeric contributions of complex mixtures was made possible.

The structural identification of individual isomers in complex mixtures is challenging as a consequence of both the complexity and the lack of authentic standards for bio-oil samples. Here, we have shown that ultrahigh-resolution MS can be used to resolve the EICs of the individual molecular compositions within complex mixtures, and the comparison of the isomeric contributions to these EICs allows the classification of isomers according to their reactivities. This, in turn, can be used to understand the differences in chemistry underlying individual chemical species and influencing upgrading strategies. Thus, it is possible to categorize isomers for elemental compositions within complex samples as highly reactive isomers (only detected before processing), low/non-reactive isomers (if detected before and after processing), and new isomeric products (isomers detected only after processing). This approach allows the simultaneous evaluation of the reactivities of thousands of chemical entities within complex mixtures such as bio-oils, providing the greatest detail to date. Furthermore, this approach can be potentially applied to the comparison of other processing or upgrading methods in order to better understand and inform future strategies for production of biofuels, for example.

Although esterification was used in this work, our innovative method can be applied to understand the reactions of bio-oils during different production or upgrading methodologies, which in turn, can help advance biofuel yields and properties.

## Supporting Information Available

TICs, KairosMS peak picking, and EICs (PDF)
Component details (XLSX)
Evolution of the mass spectra, class distribution, van Krevelen plot, and DBE verus carbon number plots with the retention time (MP4)
