# Supplementary material for: Revealing the Reactivity of Individual Chemical Entities in Complex Mixtures: the Chemistry Behind Bio-Oil Upgrading
Source: Anal Chem. 2022 May 16;94(21):7536–44. doi: 10.1021/acs.analchem.2c00261 (PMC9161218; doi:10.1021/acs.analchem.2c00261)
Supplement: Supplementary file 1 — ac2c00261_si_001.pdf [file ac2c00261_si_001.pdf]

Supporting Information

Revealing the reactivity of individual chemical entities in complex mixtures: the chemistry behind bio-oil upgrading

Diana Catalina Palacio Lozano,[a,b] Hugh E. Jones,[a,c] Remy Gavard,[c] Mary J. Thomas,[a,c] Claudia X. Ramírez,[b] Christopher A. Wootton,[a] José Aristóbulo Sarmiento Chaparro,[d] Peter O'Connor,[a] Simon E. F. Spencer,[e] David Rossell,[f] Enrique Mejia-Ospino,[b,g] Matthias Witt,[h] and Mark P. Barrow*[a]

[a]Department of Chemistry, University of Warwick, Coventry, CV4 7AL, United Kingdom

[b]Laboratorio de Espectroscopía Atómica y Molecular (LEAM), Universidad Industrial de Santander. Bucaramanga, 678, Colombia

[c]Molecular Analytical Science Centre of Doctoral Training, University of Warwick, Coventry, CV4 7AL, United Kingdom

[d]Instituto Colombiano del Petróleo (ICP-Ecopetrol), Piedecuesta, Colombia

[e]Department of Statistics, University of Warwick, Coventry, CV4 7AL, United Kingdom

[f]Department of Economics & Business, Universitat Pompeu Fabra, Barcelona 08005, Spain

[g]Centro de Materiales y Nanociencias (CMN), Universidad Industrial de Santander, Bucaramanga, 678, Colombia

[h]Bruker Daltonik GmbH, Bremen, Germany

1. Total ion chromatograms

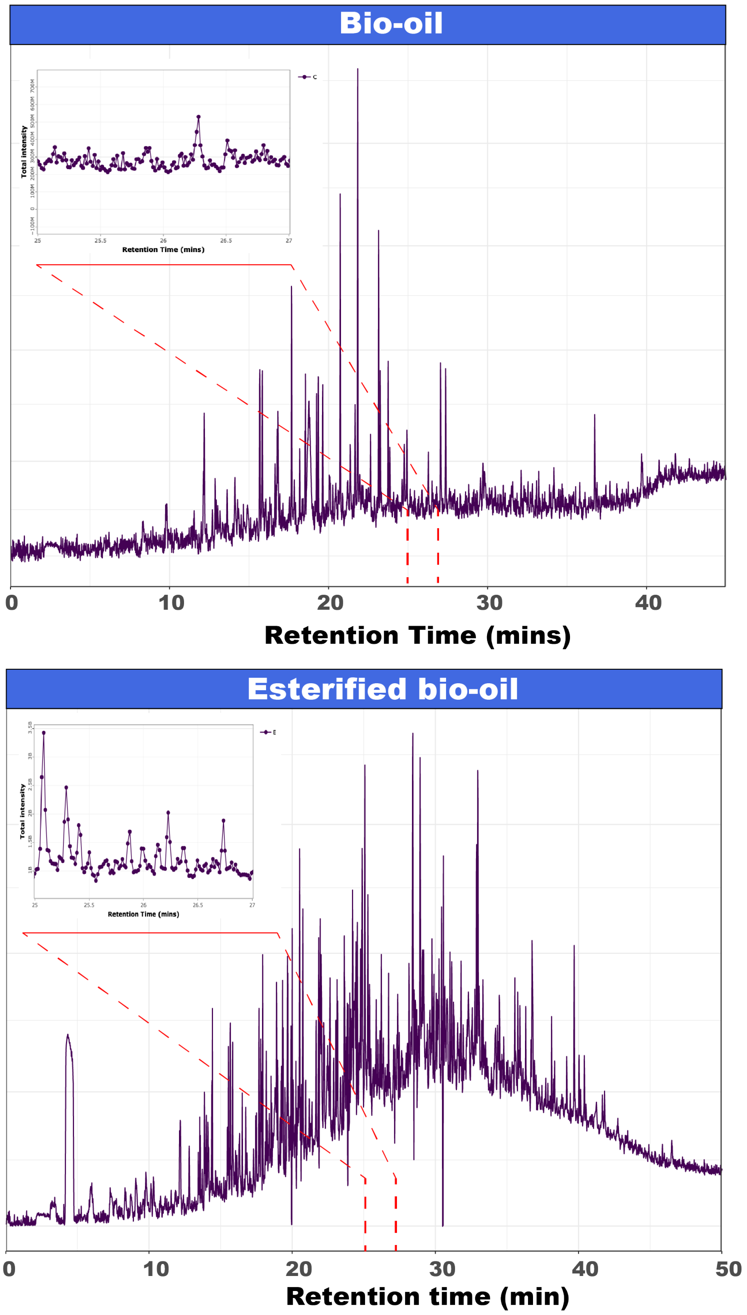

Fig. S1. Total ion chromatogram obtained from the crude bio-oil and its esterified product. The insets display the TIC obtained between 25-27 min. Each dot represents a single ultrahigh resolution mass spectrum.

2. KairosMS peak picking

Isomers are detected in a two-step process:

a) As seen in Figure S2, the local intensity maxima are detected across the EIC

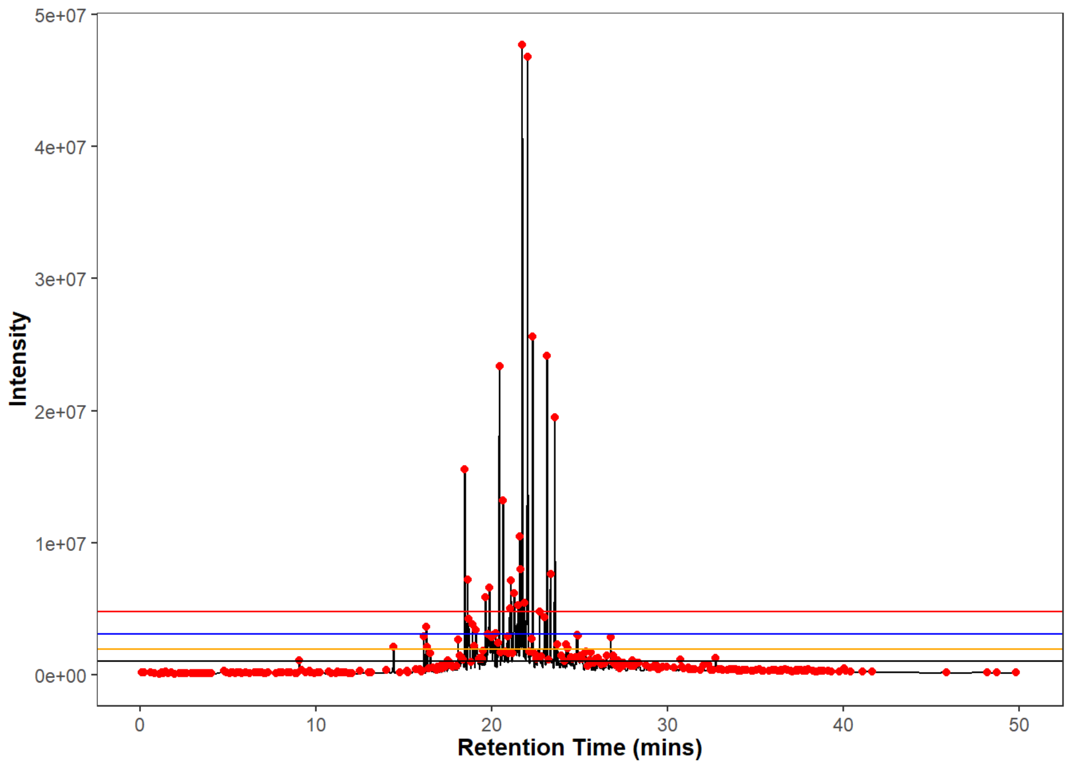

Fig. S2. Unfiltered intensity maxima.

b) In the following description, the parameters that can be modified by the user are given in *italics*. Intensity maxima are filtered according to a simple model; the noise (**blue line**) is taken to be the mean intensity across the EIC scaled by a user parameter (*noise_scalar*). The minimum allowed intensity across the EIC (**black line**), int_minimum (**red line**), is taken to be the maximum intensity multiplied by *int_scalar*. Any maxima below the threshold (**orange line**) given by; (noise + int_minimum)/ *divisor* are removed. The final peaks picked are highlighted with a red dot in Fig. S3.

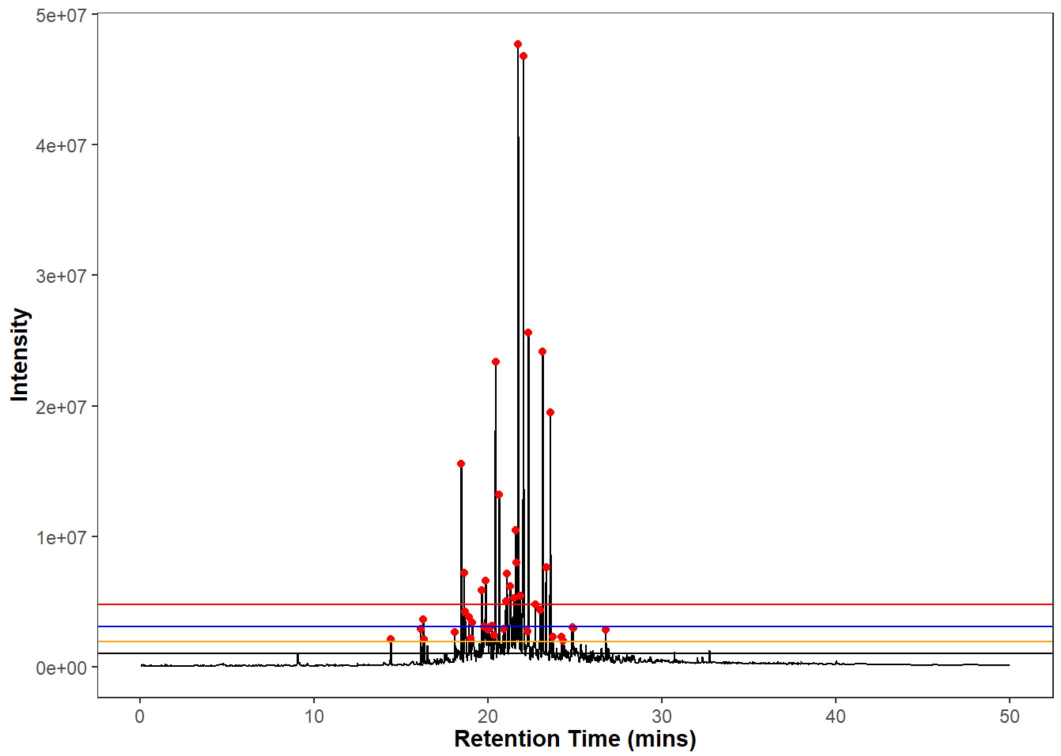

Fig. S3. Picked maxima after filtering: Black line mean intensity of the EIC, orange line threshold, blue line noise and red line minimum intensity allowed. Peaks picked are highlighted with a red dot.

3. Extracted ion chromatograms

The standard deviation, total data points, and the m/z minimum and maximum of EICs detected at m/z 227 can be seen in Table S1.

*Table S1. Extracted ion chromatograms assigned within the window at m/z 227-228. *SD: standard deviation (report in Da).*

		**Esterified bio-oil**	**Bio-oil**
227.05480	SD*	1.2 E-5	1.4E-5
$C_{10}H_{10}O_6[H]$	Total data points	1212	921
	m/z minimum	227.0548	227.0549
	m/z maximum	227.0549	227.0551
227.070168	SD	1.3E-5	1.5E-5
$C_{14}H_{10}O_3[H]$	Total data points	256	545
	m/z minimum	227.0701	227.0702
	m/z maximum	227.0702	227.0703
227.091304	SD	1.6E-5	1.3E-5
$C_{11}H_{14}O_5[H]$	Total data points	1711	1552
	m/z minimum	227.0912	227.0913
	m/z maximum	227.0914	227.0914
227.106551	SD	1.3E-5	1.5E-5
$C_{15}H_{14}O_2[H]$	Total data points	94	501

	m/z minimum<br>*m/z* maximum	227.1066<br>227.1067	227.1065<br>227.1066
227.12768<br>$C_{12}H_{18}O_4[H]$	SD<br>Total data points<br>*m/z* minimum<br>*m/z* maximum	1.8E-5<br>2012<br>227.1276<br>227.1278	2.4E-5<br>1081<br>227.1276<br>227.1278
227.164052<br>$C_{13}H_{22}O_3[H]$	SD<br>Total data points<br>*m/z* minimum<br>*m/z* maximum	2.2E-5<br>1877<br>227.1640<br>227.1642	2.6E-5<br>1434<br>227.1640<br>227.1641
227.200448<br>$C_{14}H_{26}O_2[H]$	SD<br>Total data points<br>*m/z* minimum<br>*m/z* maximum	2.1E-5<br>20902<br>227.2005<br>227.2006	1.5E-5<br>2903<br>227.2004<br>227.2005
227.236812<br>$C_{15}H_{30}O_1[H]$	SD<br>Total data points<br>*m/z* minimum<br>*m/z* maximum	1.4E-5<br>2112<br>227.2368<br>227.2370	1.1E-5<br>2899<br>227.2368<br>227.2369

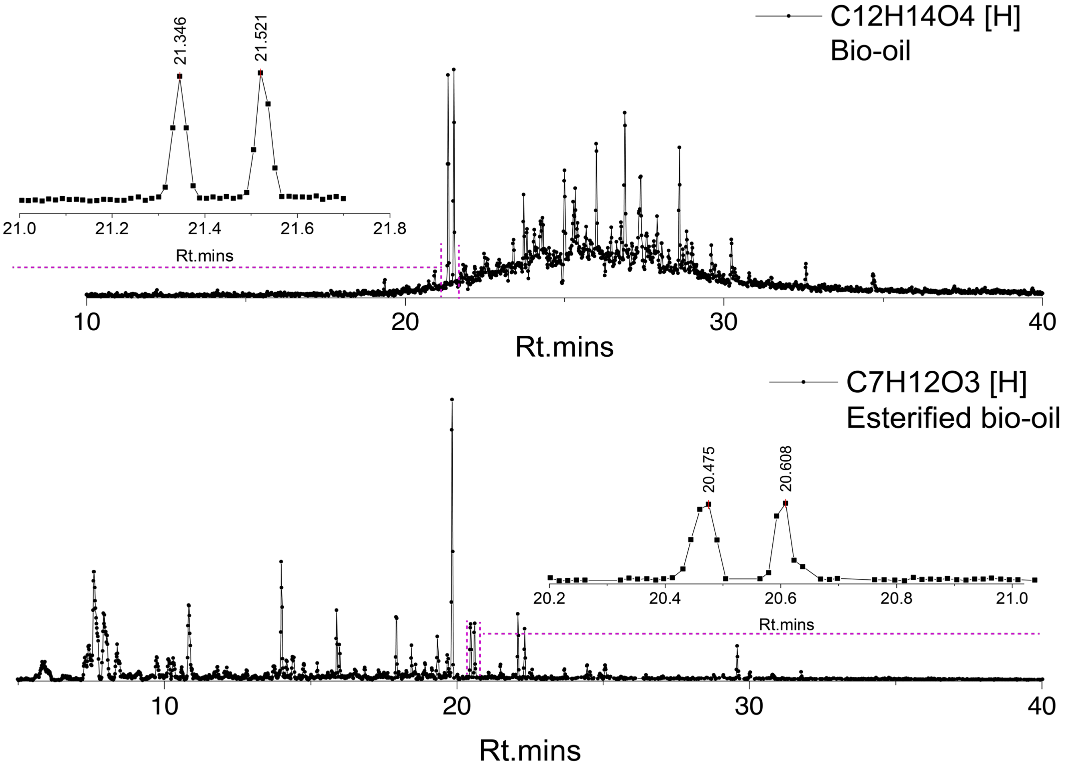

Fig. S4. Extracted ion chromatograms of the composition $C_{12}H_{14}O_4[H]$ and $C_7H_{12}O_3[H]$ detected in the bio-oil sample and the esterified sample respectively. As shown in the zoom in inserts, the fast scanning of the 7 T FTICR MS operating in 2ω detection allows a baseline separation of chemical species eluting from the GC column with a time difference of 0.175 min and 0.133 min.

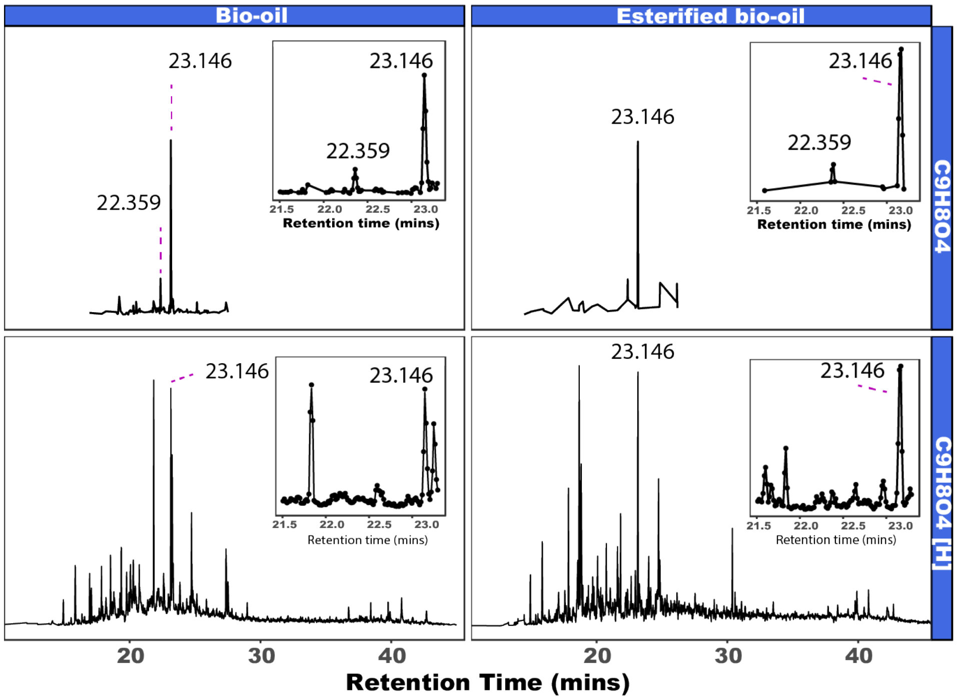

Fig. S5. Comparison of the EICs of the radical ion (top) and protonated (bottom) forms of $C_9H_8O_4$. Note the greater signal-to-noise ratio (due to greater abundance) of the protonated species. A greater number of isomers were detected as a protonated ions by APCI.

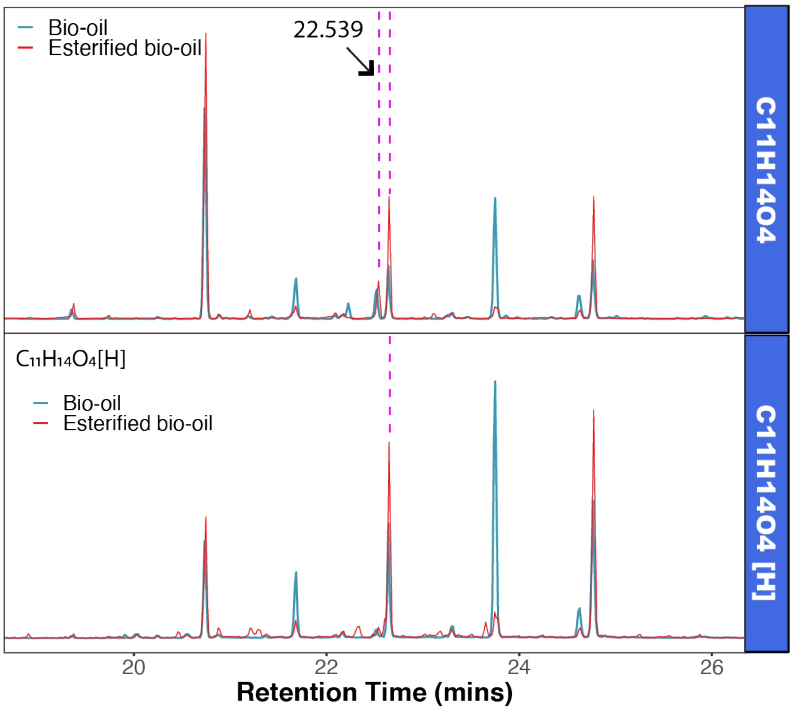

Fig. S6 Comparison of the EICs of $C_{11}H_{14}O_4$, detected as in radical ion (top) and protonated (bottom) forms. This figure shows an example of a composition detected as protonated and radical ion with high abundance. Notice the different relative abundances of the common even and odd electron ions and the unique peak detected at 22.539 min in its radical ion form.

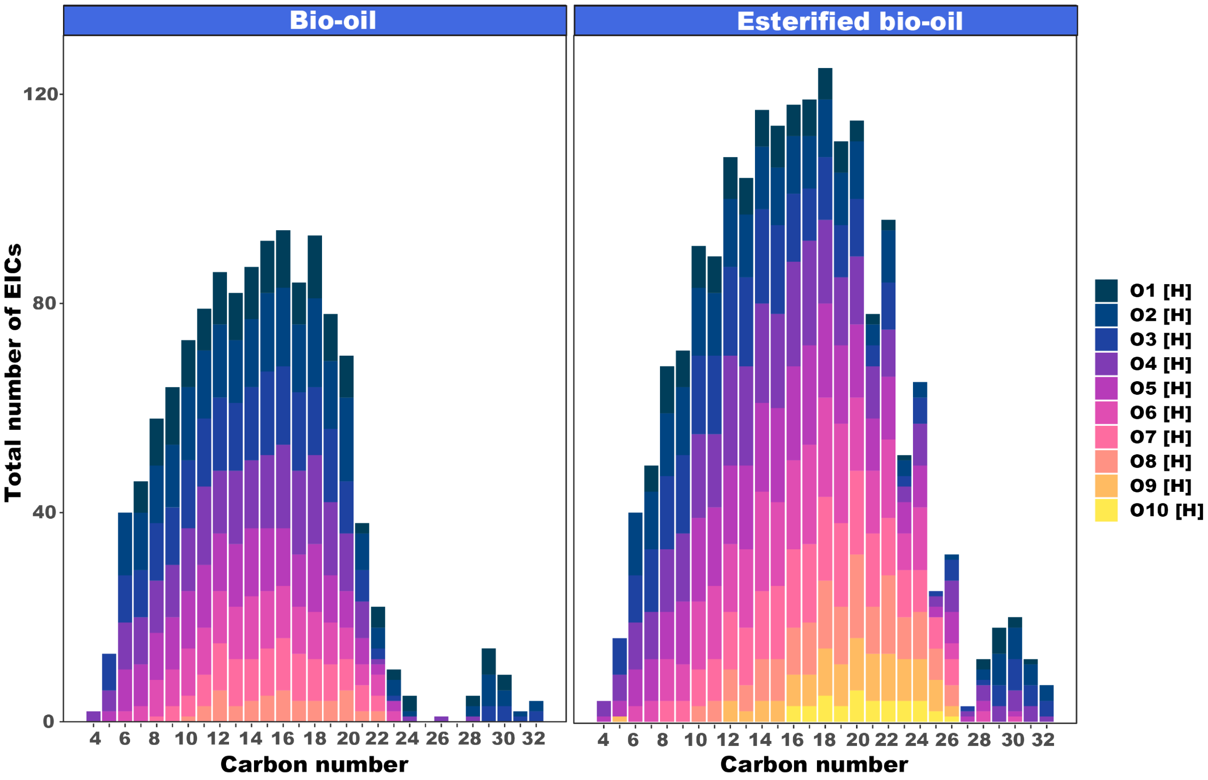

Fig. S7. Distribution of the total number of EICs detected for the bio-oil and the esterified bio-oil as a function of carbon number and heteroatom class..

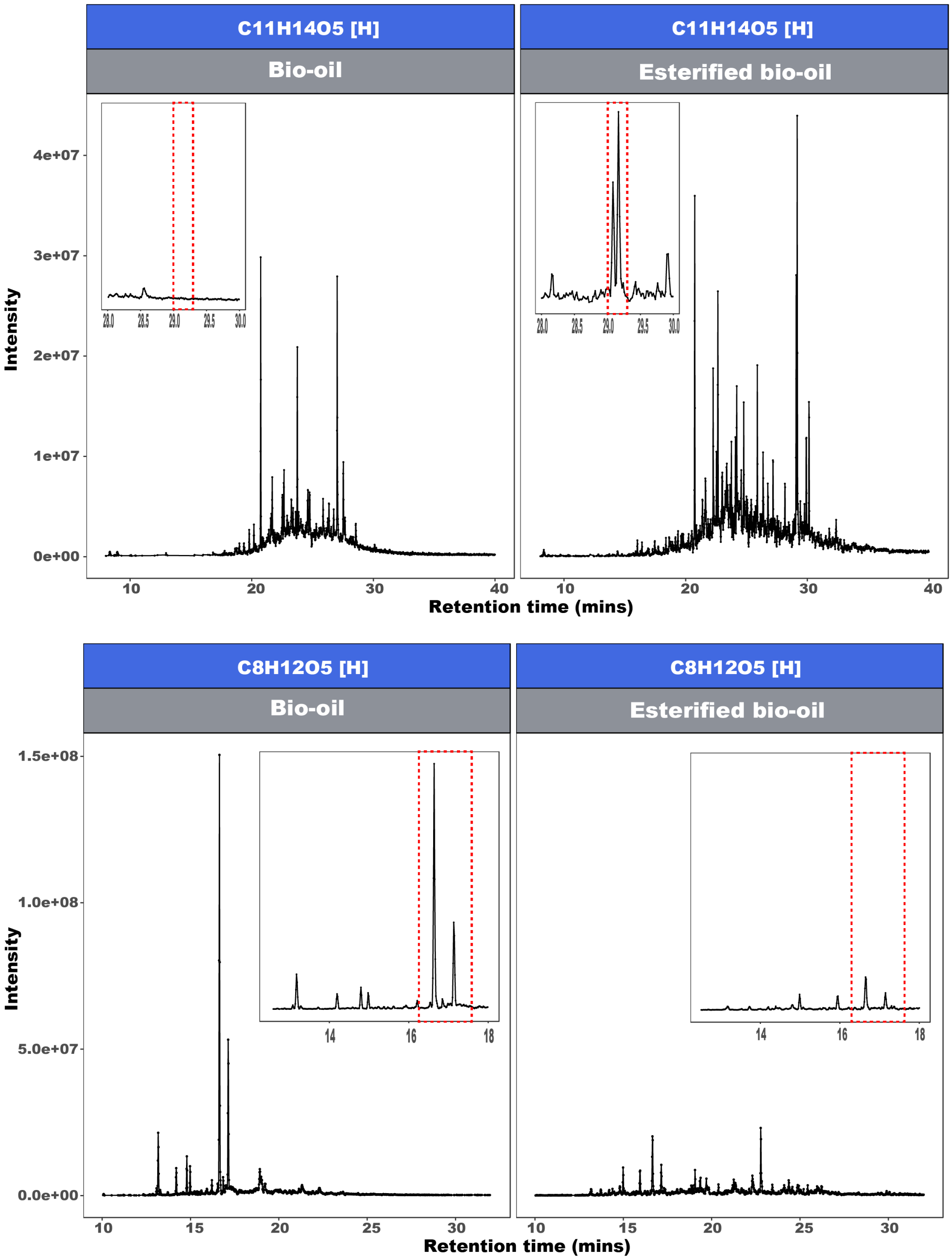

Fig. S8. Comparison of selected EICs of compositions assigned to the class O_5[H] before and after esterification. The enlarged region of the EICs highlight the selectivity of the esterification process. In the EICs corresponding to $C_{11}H_{14}O_5$[H] it is highlighted new isomers detected in the esterification sample between 29-29.5 min. This new isomers are likely species containing esters or acetals groups produced after esterification.The highlighted area of the EICs corresponding to $C_8H_{12}O_5$[H], show the lower relative abundance of isomer detected between 16-18 min, these species likely contain carboxylic acids, ketones, or aldehydes groups that were reduced after esterification.

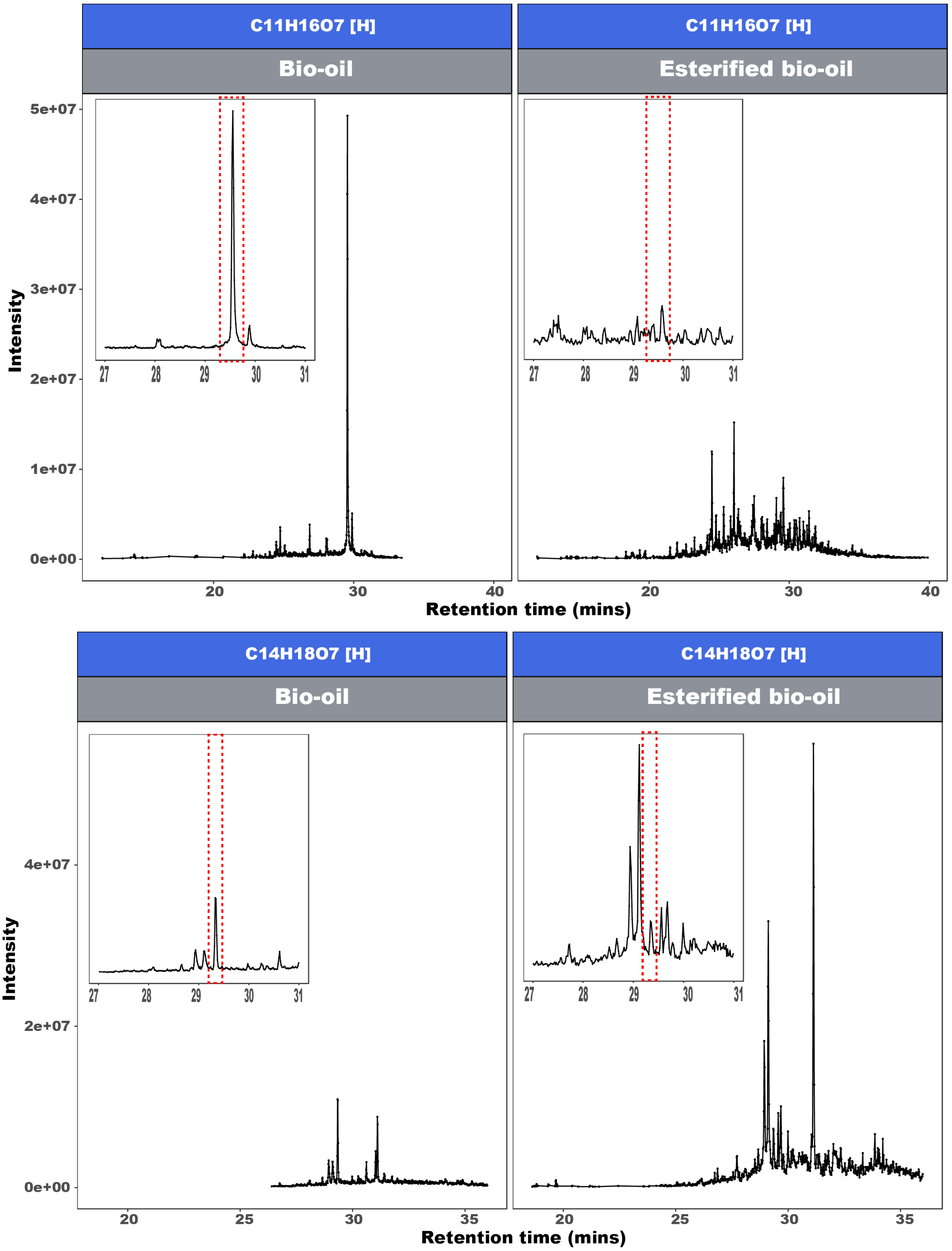

Fig. S9. EICs corresponding to some selective compositions of the class O_7[H]. In the figure it is shown a comparison of individual EICs before and after esterification. The enlarged region of the EICs highlight the selectivity of the esterification process. The low relative abundance of the isomers highlighted in the red box inidicates high reactive isomeric species towars esterification e.g. species with carboxylic acids, aldehydes, or ketone groups.

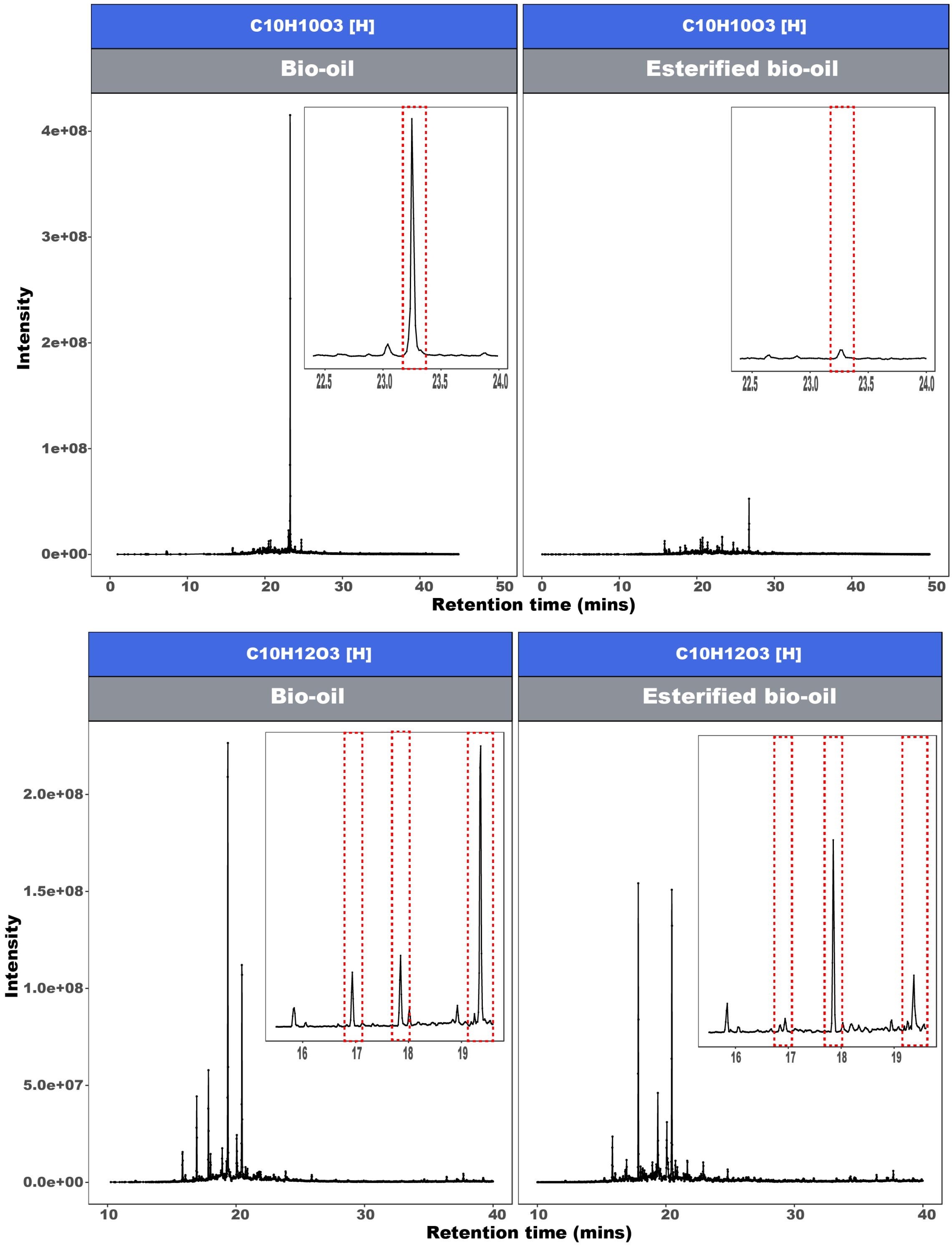

Fig. S10. Comparison of EICs corresponding of compositions of the class O_3[H] before and after esterification. The enlarged region of the EICs highlight the selectivity of the esterification process. The absence of selected signals (isomers) indicates the esterification of species containing carboxylic acids, ketones, or aldehydes functional groups that were efficiently of partially esterified (see highlighted peaks in red boxes).

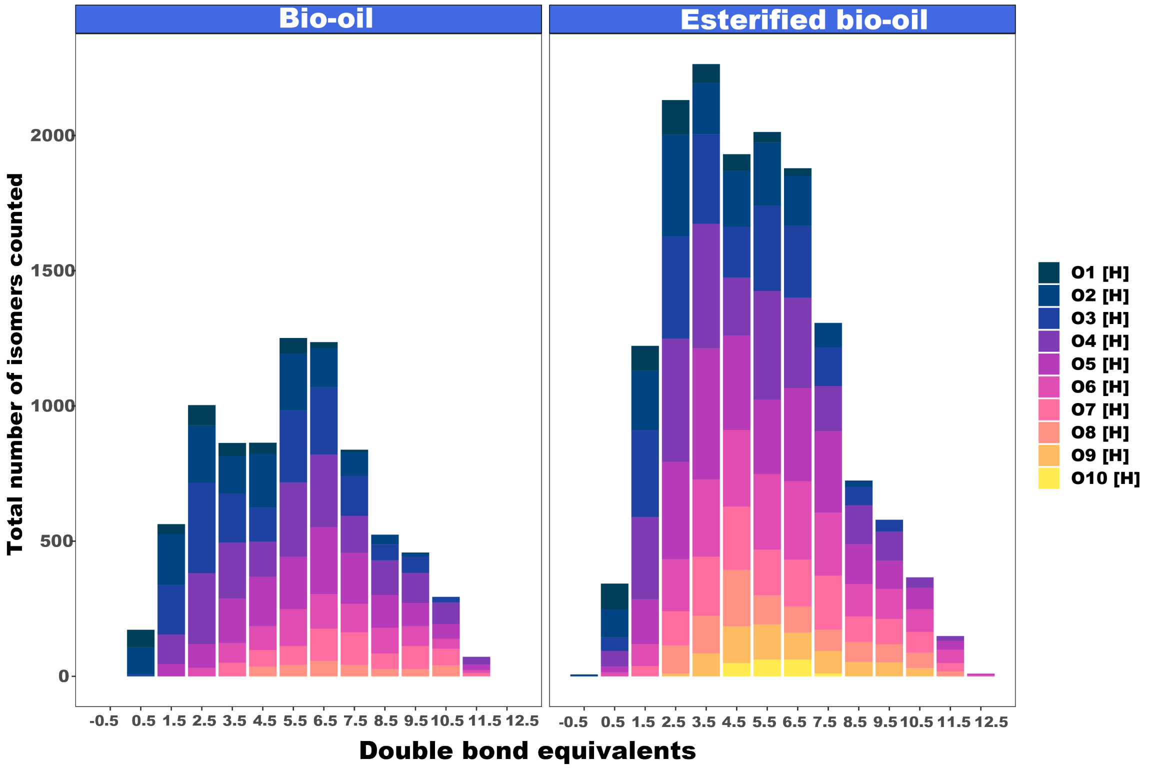

Fig. S11. Total number of isomers counted in the bio-oil and the esterified bio-oil as a function of their double bond equivalent value and heteroatom class.

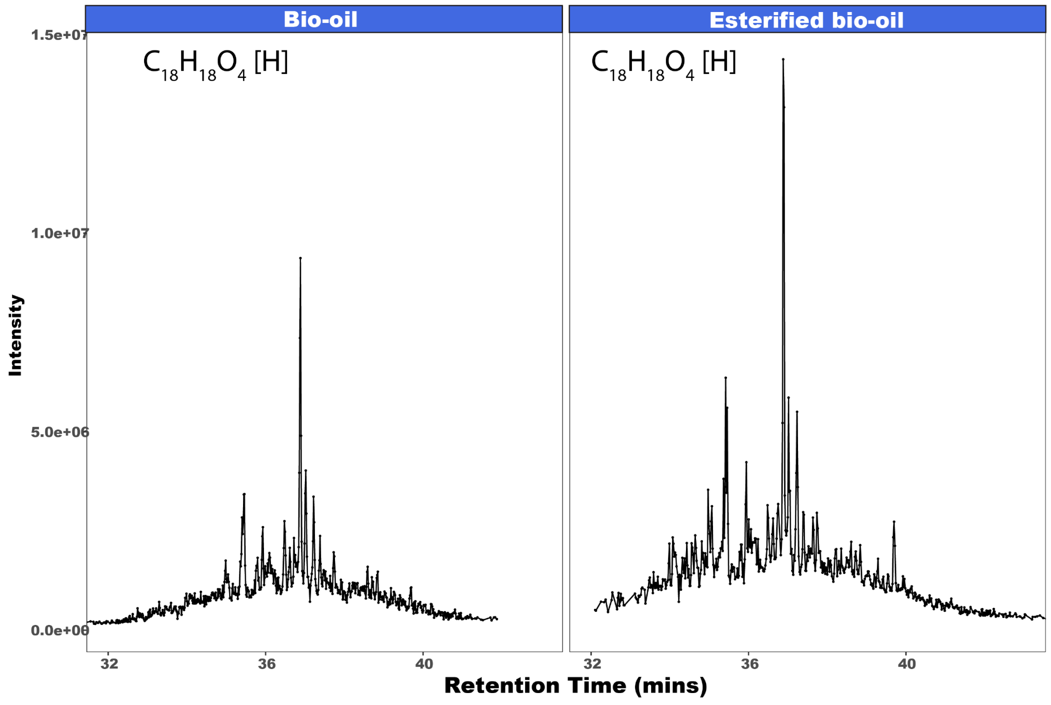

Fig. S12 Example of EICs with reduced chromatographic separation. The EICs correspond to the composition $C_{18}H_{18}O_4$[H] detected by GC-APCI-FTICR MS.

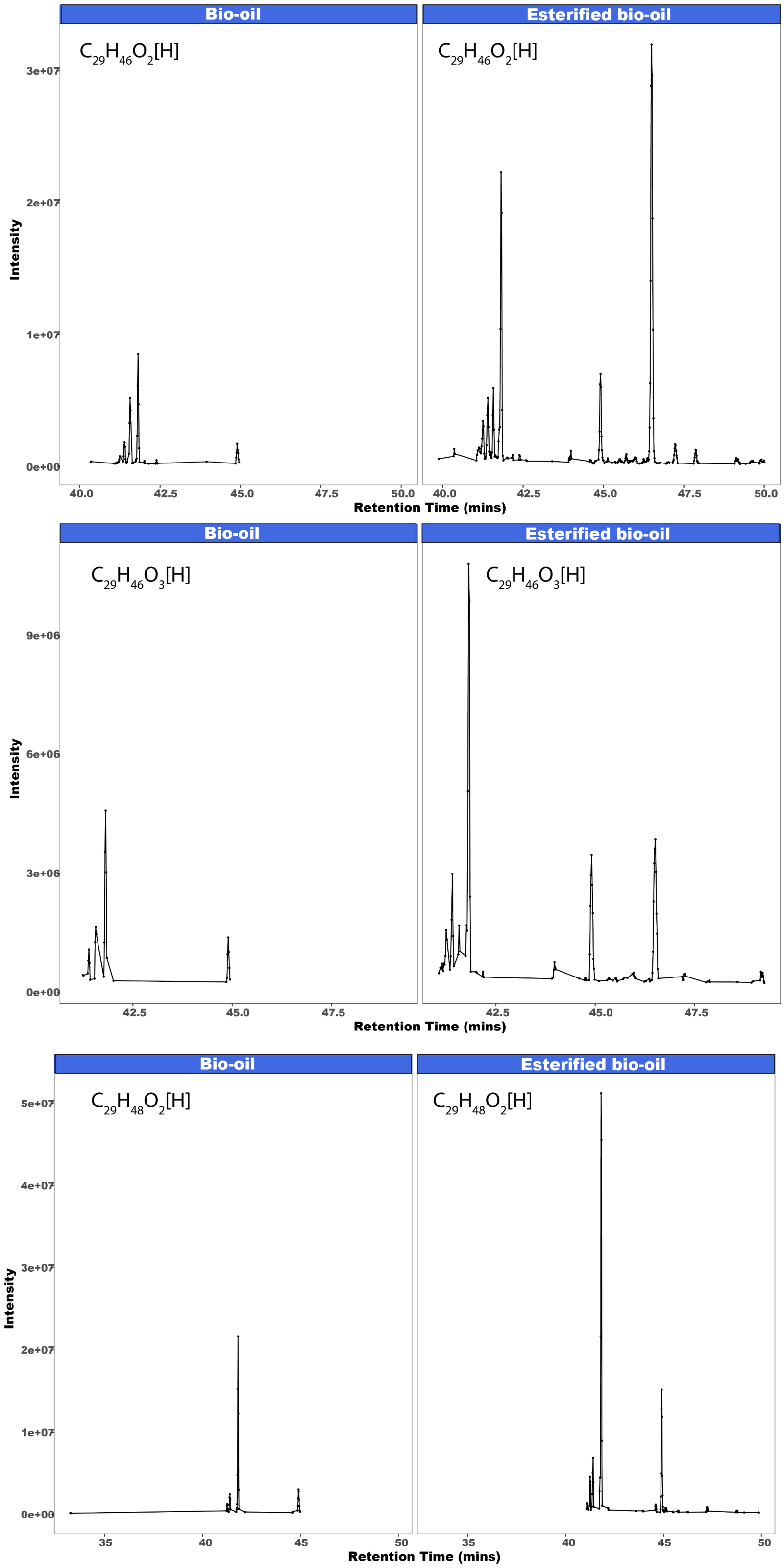

Fig. S13 EICs of compositions with high carbon number (C_{29}) detected in the bio-oil and the esterified bio-oil.Note the lower abundance detected, relative to species of lower carbon number as a result of the limitations due to the GC.

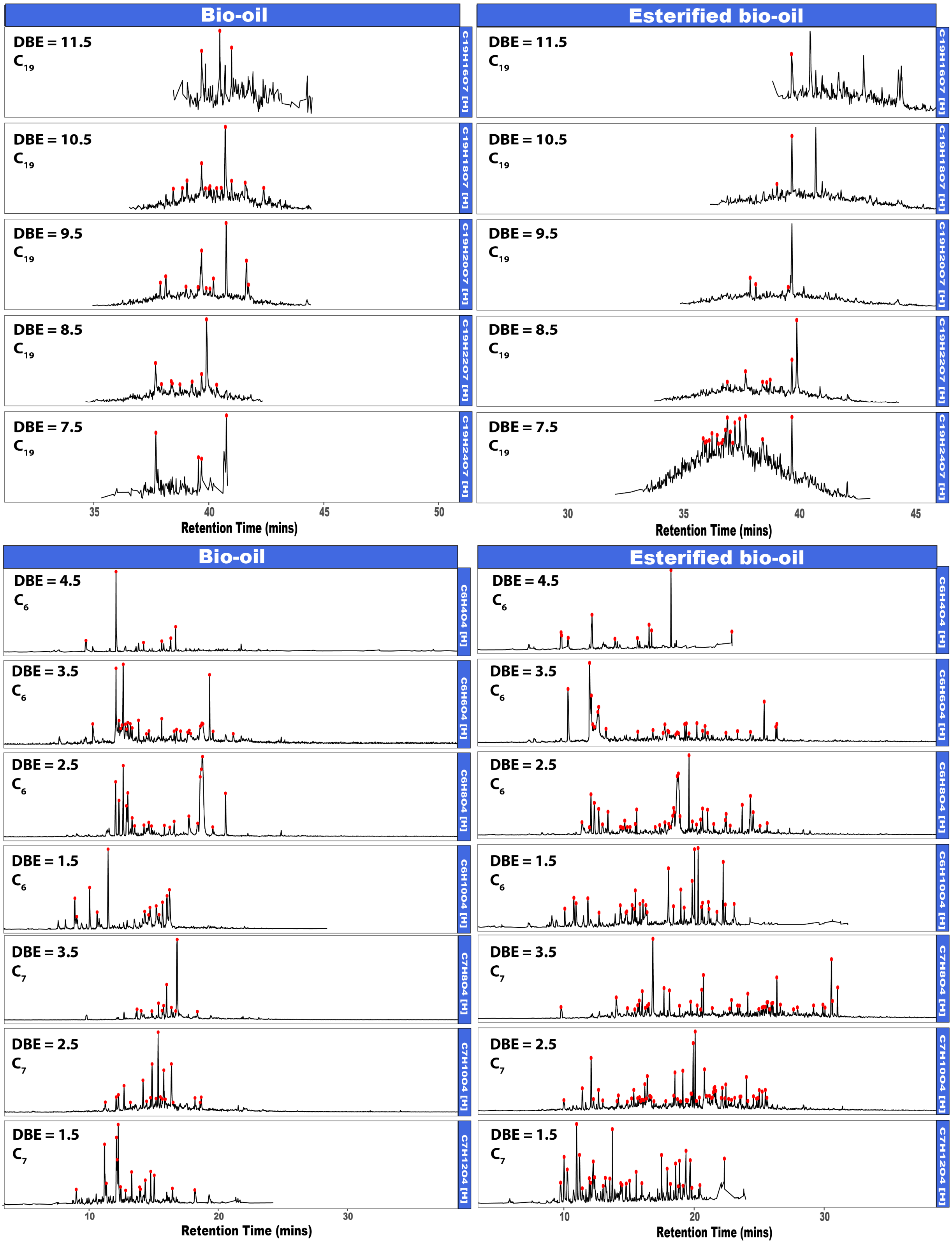

Fig. S14. Distribution of the isomeric distributions of selected EICs detected in the bio-oil and the esterified bio-oil. The red dots correspond to peaks picked under the parameters used in KairosMS.

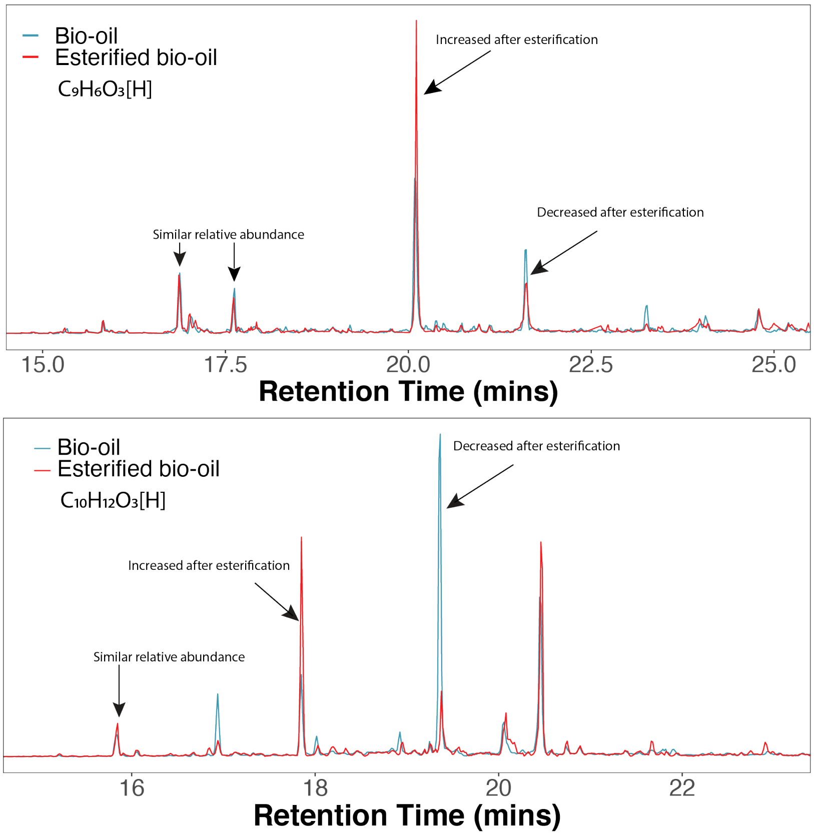

Fig. S15. EICs of low/non-reactive isomeric compositions to the esterification conditions. Moieties with a decreased intensity after esterification presumably were partially esterified, isomers with an increased intensity may correspond to products of isomers already present within the bio-oil's compositions and peaks with similar intensity showed the lowest reactivity after esterification.

Table S2. Total number of isomers observed per class. Highly reactive isomers are detected only in the bio-oil sample. Low/non-reactive are isomers detected in the bio-oil and the esterified bio-oil, and new isomeric products are isomers detected only in the esterified bio-oil.

Classes	Highly reactive isomers		Low/non-reactive isomers		New isomeric products		
	Number of isomers	%	Number of isomers	%	Number of isomers	%	Total isomers per class
$O_1[H]$	121	20.3	175	29.4	300	50.3	596
$O_2[H]$	595	30.6	516	26.6	831	42.8	1942
$O_3[H]$	863	34.5	532	21.2	1110	44.3	2505
$O_4[H]$	762	27.9	654	23.9	1317	48.2	2733
$O_5[H]$	476	21.6	576	26.1	1153	52.3	2205
$O_6[H]$	281	18.8	304	20.4	906	60.8	1491
$O_7[H]$	331	31.2	243	22.9	488	46	1062
$O_8[H]$	151	34.1	69	15.6	223	50.3	443
$NO_1[H]$	4	36.4	7	63.6	0	0	11
$NO_2[H]$	15	34.1	19	43.2	10	22.7	44
$NO_3[H]$	53	30.3	44	25.1	78	44.6	175
$NO_4[H]$	85	34.4	44	17.8	118	47.8	247
$NO_5[H]$	81	36.5	40	18	101	45.5	222
$NO_6[H]$	40	35.1	16	14	58	50.9	114
Total	3858	30.4	3239	26.3	6693	43.3	13790
